# Protocol for single-base quantification of RNA m5C by pyrosequencing

**DOI:** 10.1016/j.xpro.2025.104099

**Published:** 2025-09-18

**Authors:** Akin Cayir, Yvonne Böttcher

**Affiliations:** 1 EpiGen, Medical Division, Akershus University Hospital, 1478 Lørenskog, Norway; 2 Department of Clinical Molecular Biology, EpiGen, Institute of Clinical Medicine, University of Oslo, 1171 Oslo, Norway

**Keywords:** Cell Biology, Single Cell, Genetics, Genomics, Sequencing, Molecular Biology

## Abstract

5-methylcytosine (m5C) is a common RNA modification found in both coding and non-coding RNAs. Here, we present a protocol for RNA-m5C-pyroseq for quantitative, single-nucleotide resolution analysis of RNA methylation. We describe steps for designing primers for the target region of RNA, performing bisulfite conversion of RNA, and using pyrosequencing to measure RNA methylation in cytosine. We then detail procedures for all the steps, including RNA isolation, bisulfite conversion, cDNA synthesis, PCR, gel electrophoresis, and pyrosequencing.

## Highlights

• Steps for single-nucleotide resolution analysis of RNA m5C
• Guidance on primer design using PyroMark Assay Design 2.0
• Instructions for preparing RNA for pyrosequencing analysis

## Abstract (editor highlights)

Publisher’s note: Undertaking any experimental protocol requires adherence to local institutional guidelines for laboratory safety and ethics.

## Graphical abstract

**Figure:**
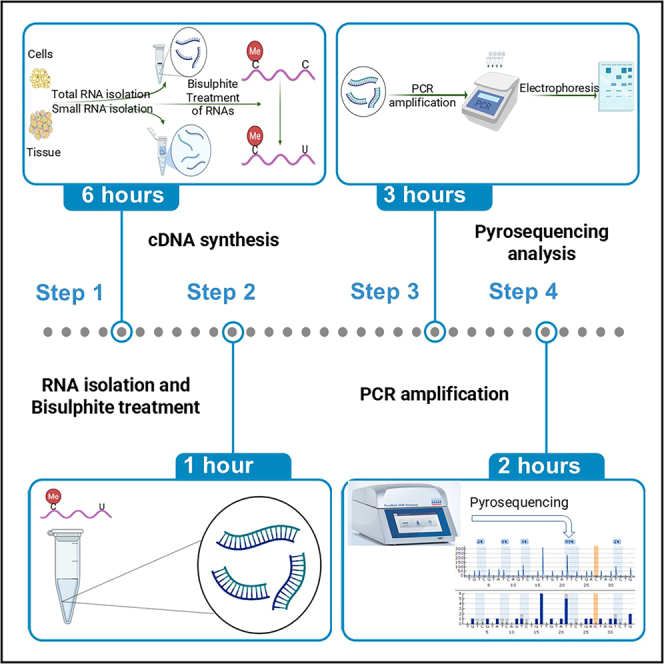

## Before you begin

### Innovation

Various RNA species within human cells contain a multitude of modified bases, with over 160 reported modifications documented in eukaryotes to date.^1^ One prevalent modification is 5-Methylcytosine (m5C), resulting from the addition of a methyl group to the cytosine base. While the presence of m5C in human messenger RNAs (mRNAs) is recognized,^2^ non-coding RNAs (ncRNAs), including transfer RNAs (tRNAs), and ribosomal rRNAs (rRNAs), which are highly critical components of protein synthesis, are acknowledged for harboring m5C at specific loci.^3^ More specifically, a transcriptome-wide study demonstrated that methylated cytosine has been identified in more than 10,275 candidate sites in mRNAs, tRNAs, and other types of non-coding RNAs.^2^

In order to determine the existence of m5C modification in various RNAs, initial studies have used chromatography based approaches such as thin-layer and liquid chromatograph, a combination of mass spectrometry and liquid chromatography has been used to quantify individual RNA modification including m5C,^4^ which was based on the digestion of RNAs to nucleosides.^5^ These approaches provide high accuracy of quantity of modified base in bulk RNAs or bulk of a specific type of RNAs (e.g., in mRNAs or in tRNAs), lacking single nucleotide resolution knowledge. On the other hand, sequencing based methods such as RNA-bisulfite sequencing,^6^ methylated-RNA-immunoprecipitation,^7^ 5-azacytidine–mediated RNA immunoprecipitation (Aza-IP)^8^ and miCLIP^9^ have been used to map the m5C in RNAs, which provide the presence or the distribution of the m5C in over RNAs. Such approaches utilize enrichment or chemical treatment of RNA, which facilitate the detection of RNA m5C modification.

Here, we developed a method, called RNA-m5C-pyroseq, to quantify m5C in 28S rRNA and tRNAs in a single-base locus-specific resolution to perform fast and easy candidate gene analyses. Our methodology is composed of an integration of bisulfite treatment with pyrosequencing technology, the latter which has been successfully used for DNA m5C quantification. Our technique substantially reduces the required RNA amount as starting material, provides single-base locus-specific resolution, and quantifies modification levels at specific positions within RNA molecules. As a result, this adapted approach holds high potential for wide-ranging applications in various research fields such as large human cohorts for high throughput analyses.

## Key resources table

**Table:** 

REAGENT or RESOURCE	SOURCE	IDENTIFIER
**Biological samples**		

Preadipocytes derived from subcutaneous adipose tissue (SAT)	N/A	N/A

**Chemicals, peptides, and recombinant proteins**		

RNaseZap RNase decontamination solution	Thermo Fisher Scientific	Cat#AM9780
Liquid nitrogen	N/A	N/A

**Critical commercial assays**		

RNeasy MinElute cleanup kit	QIAGEN	Cat#74204
QIAshredder	QIAGEN	Cat#79656
DNA-free DNA removal kit	Thermo Fisher Scientific	Cat#AM1906
SuperScript III reverse transcriptase kit	Invitrogen	Cat#18080093
RNAseOUT	Thermo Fisher Scientific	Cat#10777019
GoTaq hot start green master mix	Promega	Cat#M5122
EZ RNA Methylation kit	Zymo Research	Cat#R5002
DNA LoBind tubes	Eppendorf (1.5 mL)	Cat#30108051
PyroMark Q48 magnetic beads	QIAGEN	Cat#974203
PyroMark Q48 absorber strips	QIAGEN	Cat#974912
PyroMark Q48 advanced CpG reagents	QIAGEN	Cat#974022
dNTP mix (10 mM each)	Thermo Fisher Scientific	Cat#R0192
PyroMark Q48 discs	QIAGEN	Cat#974901
Qubit dsDNA HS kit (100)	Thermo Fisher Scientific	Cat#Q32851

**Oligonucleotides**		

Random hexamers	Thermo Fisher Scientific	Cat#N8080127
*28S rRNA*, F: 5′GGGGTTTTAYGATTTTTTTGATTTTTTGGG3′	Thermo Fisher Scientific	N/A
*28S rRNA*, R: biotin-5′CCAACTCACRTTCCCTATTAATAAATAAAC 3′	Thermo Fisher Scientific	N/A
*28S rRNA,* sequencing: 5′ GTGGYGGTTAAGYGTTTATA 3′	Thermo Fisher Scientific	N/A
*tRNA-ASP,* F: 5′ TAGTATAGTGGTGAGTATT 3′	Thermo Fisher Scientific	N/A
*tRNA-ASP,* R: biotin-5′ CTCCCCATCAAAAAATCA 3′	Thermo Fisher Scientific	N/A
*tRNA-ASP,* sequencing: 5′ TAGTATAGTGGTGAGTATT 3′	Thermo Fisher Scientific	N/A

**Other**		

PyroMark Q48 Autoprep	QIAGEN	Cat#9002471
Vortex	IKA	N/A
Centrifuge with a swing-out rotor suited for 50-mL conical tubes	Thermo Fisher Scientific	Microfuge X3R
Tabletop centrifuge with a swing-out rotor for 1.5-mL tubes	VWR	Microstar 21R
Centrifuge with a swing-out rotor suited for 15-mL conical tubes	Thermo Fisher Scientific	Microfuge X3R
Thermomixer	Eppendorf 1.5 mL	N/A
Agarose gel electrophoresis apparatus.	Bio-Rad	N/A
Thermal cycler	Eppendorf	Mastercycler X50i
Laminar flow hood (LAF) bench	Herasafe	Cat#2030

***Note:*** The primers listed in this table were used to generate the pyrosequencing data for 28S rRNA and tRNA-Asp. The forward and reverse primers for 28S rRNA were obtained from the Zymo Research EZ RNA Methylation Kit protocol. The forward and reverse primers for tRNA-Asp were sourced from Amort et al.^10^ The sequencing primers were designed by the authors.

***Note:*** Along with the primers listed in the table, we also designed additional primers using PyroMark Assay Design 2.0. The procedure for generating these primers is described in the ‘assay design for pyrosequencing’ section, and the generated primers have been tested and validated for the protocol.

## Materials and equipment

### Buffers

***Alternatives:*** All the reagents used in this protocol can be replaced with the same function from different suppliers.

### Chemical solution

• 70% EtOH (70 mL of 100% EtOH, to 100 mL with ddH2O) (Room temperature).
• RNA Wash Buffer (48 ml of 100% ethanol (52 ml of 95% ethanol), to the 12 ml RNA Wash Buffer concentrate) (Room Temperature).
• 50 nM of random hexamer (10 μL of 0.5 μM/μL, to 90 μL with RNase-free water) (−20°C).
• 100 μM of primers (adding DNase/RNase-free water as indicated in instruction) (−20°C).
• Prepare 10 μM of primers (10 μL of 100 μM primers, to 90 μL of RNase-free water) (−20°C).

**CRITICAL:** Use annealing buffer to prepare sequencing primer for pyrosequencing.

### Equipment

• PyroMark Q48 Autoprep.

***Alternatives:*** PyroMark Q24 and PyroMark Q96 Systems.

• HERASAFE LAF bench.

***Alternatives:*** Any LAF bench is suitable to work during FA.

• VWR centrifuge with a swing-out rotor for 1.5-mL tubes.

***Alternatives:*** Any centrifuge is suitable for 1.5 mL tubes with cooling system.

• Thermo Fisher Scientific Centrifuge with a swing-out rotor suited for 15-mL and 50-mL conical tubes.

***Alternatives:*** Any centrifuge is suitable for 1.5 mL tubes with cooling system.

• IKA Vortex.

***Alternatives:*** Any vortex is suitable.

• BIO-RAD Agarose gel electrophoresis apparatus.

***Alternatives:*** Any gel system.

• Eppendorf Thermal Cycler.

***Alternatives:*** Any thermal cycler.

## Step-by-step method details

### Isolation of total and small RNAs

**Timing: 90 min**

This protocol is developed to measure methylation level at a single nucleotide level in human 28S rRNA and tRNA-Asp (anticodon GTC). It is optimized for pre-adipocytes derived from subcutaneous adipose tissue (SAT). In this step, we isolate total RNA to measure m5C in 28S rRNA and small RNA-enriched fraction to measure m5C in human tRNA-Asp.

### Processes for total RNA isolation

1. Shock-freeze the cells by placing the plate into liquid nitrogen for 1–2 min.
  a. Place on normal on ice.
  b. Add 350 μl QIAzol/Well and collect cell lysate.
  c. Transfer lysates into two 1.5 ml tubes and mix by pipetting.
2. Transfer lysates into QIAshredder spin column.
  a. Centrifuge for 2 min at full speed (>10,000 rpm) at room temperature (RT).
3. Place lysate on the bench top for 3–5 min at RT.
4. Add 70 μl chloroform and shake for 15 s after closing tube.
5. Place on bench top and incubate for 2–3 min at RT.
6. Centrifuge at 12,000 g for 30 min at 4°C.
7. Transfer the aqueous phase containing the RNA to a new Tube (∼200 μl).
8. Add 1 Volume 70% EtOH (∼200 μl).
  a. Mix by pipetting.
9. Transfer up to 700 μl of the sample to a RNeasy spin column.
10. Close lid and centrifuge for 15 s at >8,000 g (>10,000 rpm).
  a. Discard flow through and place spin column back to collection tube.
11. Add 700 μl Buffer RW1 to the RNeasy spin column.
  a. Centrifuge for 15 s at >8,000g (>10,000 rpm).
  b. Discard flow through and place spin column back to collection tube.
12. Add 500 μl Buffer RPE to the RNeasy spin column.
  a. Centrifuge for 15 sec at >8,000g (>10.000 rpm).
  b. Discard flow through.
13. Repeat wash with 500 μl RPE.
  a. Centrifuge for 2 min at >8,000 g (>10,000 rpm).
  b. Discard flow through.
14. Centrifuge spin column at full speed for 1 min to remove residual EtOH.
15. Place spin column in a new 1.5 ml collection tube.
  a. Add 40 μl of RNase free water directly to the membrane of the spin column.
  b. Incubate for 1 min at RT.
  c. Centrifuge for 1 min at >8,000 g (>10,000 rpm) to elute RNA.
16. Measure RNA concentration.
  a. Store total RNA samples in −80°C.

### Processes for small RNA-enriched fraction

17. After step 7 of total RNA extraction procedure,
  a. Transfer the upper aqueous phase to a new reaction.
  b. Add 1 volume of 70% ethanol (usually 350 μl).
  c. Mix thoroughly by vortexing.
18. Pipet the sample into a RNeasy Mini spin column placed in a 2 ml collection tube.
19. Centrifuge at ≥8000 x g (≥10,000 rpm) for 15 s at room temperature (15°C–25°C).

**CRITICAL:** Flow-through contains small RNAs.

20. Add 450 μl of 100% ethanol (0.65 volumes) to the flow-through from step 19 by mix vortexing.
21. Pipet 700 μl of the sample into a RNeasy MinElute spin column centrifuge for 15 s at ≥8000 x g (≥10,000 rpm) at room temperature.
  a. Discard the flow-through.
22. Pipet 500 μl Buffer RPE into the RNeasy MinElute spin column and centrifuge for 15 s at ≥8000 x g (≥10,000 rpm).
  a. Discard the flow-through.
23. Add 500 μl of 80% ethanol to the RNeasy MinElute spin column and centrifuge for 2 min at ≥8000 x g (≥10,000 rpm).
  a. Discard the flow-through and the collection tube.
24. Place the RNeasy MinElute spin column into a new 2 ml collection tube.
  a. Centrifuge for 5 min at ≥8000 x g (≥10,000 rpm).
25. Place the RNeasy MinElute spin column into a 1.5 ml collection tube.
  a. Pipet 14 μl of RNase-free water onto the spin column membrane.
  b. Centrifuge for 1 min at ≥8000 x g (≥10,000 rpm) to elute the small RNAs-enriched fraction.

***Note:*** Small RNAs and long RNAs (>200 bp) can be isolated from the same sample separately.

***Note:*** Work in the hood.

### DNase I treatment

**Timing: 45 min**

Here, we treat RNA samples with DNase I DNA-free DNA Removal Kit to degrade DNA from RNA samples.

26. Add 0.1 volume 10X DNase I buffer and 1 μL of rDNase I to the RNA and mix gently.
27. Incubate at 37°C for 30 min.
28. Add 2 μL of resuspended DNase inactivation reagent and mix well.
29. Incubate 2 min at room temperature and mixing occasionally.
30. Centrifuge at 10,000 x g for 1.5 min and transfer the RNA to a new tube.
31. Keep the rDNase I treated RNA at −80°C for the next bisulfite treatment step.

### Bisulfite treatment

**Timing: 5 h for 28S rRNA assay and 150 min for tRNA assay**

Here, we perform bisulfite treatment of 1 μg of total and 1 μg of small RNAs. Bisulfite treatment leads to the conversion of unmethylated cytosines into uracils while methylated cytosines remain unchanged. We follow the protocol for the bisulfite conversion of total RNAs or small RNAs by EZ RNA Methylation Kit with minor modifications.

32. Add 130 μl of RNA conversion reagent to a total 20 μl of RNA from step 31 containing 1 μg of RNA in a PCR tube.

**CRITICAL:** If the RNA sample volume is below 20 μl, add DNase/RNase-free water to reach a final volume of 20 μl.

33. Mix the sample by gentle pipetting and then spin briefly.

***Note:*** Pipet at least ten times on ice-cold.

34. Divide each sample into two tubes, 75 μl of each,
  a. Place the tubes in thermal cycles by following the cycle (Table 1).

**Table 1:** Bisulfite conversion conditions of RNAs

Cycle number	Temperature	Time
1	70°C (Denaturation stage)	5 min
1	54°C	45 min
–	4°C	Up to 20 h, optional

***Note:*** Lid temperature of thermal cycler should be 70°C during bisulfite treatment of RNA.

**CRITICAL:** Apply 5 cycles (70°C and 54°C cycles sequentially) to ensure for bisulfite conversion in case of 28S rRNA.

**CRITICAL:** Apply 3 cycles in case of tRNAs due to complexity.^11^

35. After thermal cycle step, add 250 μl of RNA binding buffer to the Zymo-Spin IC column with collection tube at RT.
36. Load the sample (two tubes) from step 34 into the Zymo-Spin IC column.
  a. Mix by gentle pipetting with RNA binding buffer at RT.

***Note:*** Pipet at least ten times on ice-cold.

37. Add 400 μl of 100% of ethanol to the mixture and RNA sample + binding buffer in the column at RT.
38. Close the cap and immediately mix the column by inverting several times at RT.
39. Centrifuge at full speed (>10,000 g) for 30 seconds and discard the flow-through at RT.
40. Add 200 μl of RNA wash buffer to the column and centrifuge at full speed (>10,000 g) for 30 seconds at RT.
41. Add 200 μl of RNA sulfonating buffer to the column,
  a. Put in a heater at 27°C for 30 minutes.
  b. After incubation, centrifuge the tubes at full speed for 30 seconds.
  c. Discard the flow-through.
42. Add 400 μl of RNA wash buffer to the column,
  a. Centrifuge it at full speed for 30 seconds.
  b. Repeat the wash step once more at RT.
43. Put Zymo-Spin IC column containing converted RNA in a new collection tube,
  a. centrifuge at full speed for 2 minutes at RT.
44. Add 20 μl of DNase/RNase-free water to the column and let stand for 1 minutes at RT.
45. Centrifuge at full speed for 30 seconds at RT.
46. Placed bisulfite converted RNA at −80°C or immediately use to synthesize cDNA.

***Note:*** Working with 10–12 samples in an experiment are recommended. Large numbers of samples require more time, which may result in low concentration of bisulfite-converted RNA.

### cDNA synthesis from bisulfite-treated RNA

**Timing: 90 min**

Here, we synthesize cDNA from bisulfite converted RNAs. We use cDNA for PCR amplification in the next step.

47. Add the following components to >200 ng of bisulfite converted RNA with the components in Table 2.

**Table 2:** Components for first stage of cDNA synthesis

Reagents	Amount
5 mM dNTPs	4
50 nM random hexamers	1
Bisulfite converted RNA	>200 ng
RNase free water	Variable
Total	13
48. Incubate the mixture in a thermal cycler at 70°C for 5 min.
49. Place the samples in ice for 1 min as soon as possible.
50. Add the reagents in Table 3 when the samples are on the ice.

**Table 3:** Components for second stage of cDNA synthesis

Reagents	Reagents
SuperScript III Reverse Transcriptase	1
RNaseOUT (40 U/μl)	1
DTT (100 mM)	1
5X First-strand buffer	4
Total	20
51. Place the samples in thermal cycles by following the condition below (Table 4).
  a. Place the cDNA at −20°C.

**Table 4:** Conditions for cDNA synthesis

Cycle number	Temperature	Time
1	25°C	5 min
1	50°C	50 min
1	75°C	15 min
–	4°C	Up to 20 h, optional

### Assay design for pyrosequencing

This section outlines each step of two assays for pyrosequencing. These steps include selected sequences of the human 28S rRNA/tRNA, sequences after bisulfite conversion, sequences of cDNA obtained from bisulfite-treated RNA, and primer sets.

### Assay design for *Homo sapiens* (human) 28S rRNA

**Timing: 60 min**

Here, we present all the details about how to design an assay to measure m5C in a specific region of 28S rRNA. We measure the methylation level at 4 CpG sites available in human 28S rRNA. One of these CpG sites is heavily methylated (as ∼100%), reported previously.^2^^,^^4^ Thus, we select the heavily methylated CpG site as positive control to test the protocol.

52. Obtain sequence of human 28S rRNA from GenBank accession with accession number (NR_003287).
53. Determine the region of interest that includes methylated cytosines.

***Note:*** The selected region in this protocol for pyrosequencing includes a CpG site (in the 4^th^ position) with 100% methylation as positive control.

***Note:*** In addition, the selected region for pyrosequencing includes three additional CpG sites (first three CpGs) for that we also measure m5C level.

**5′**GGGGCCUCACGAUCCUUCUGACCUUUUGGGUUUUAAGCAGGAGGUGUCAGAAAAGUUACCACAGGGAUAACUGGCUUGUGGCGGCCAAGCGUUCAUAG**CG**A**CG**U**CG**CUUUUUGAUCCUU**CG**AUGUCGGCUCUUCCUAUCAUUGUGAAGCAGAAUUCACCAAGCGUUGGAUUGUUCACCCACUAAUAGGGAACGUGAGCUGGGUUUAGAC **3′**

54. Determine the sequence after bisulfite conversion of 28S rRNA.

***Note:*** See an example of human 28S rRNA after bisulfite conversion.

**5′**GGGGUUUUAUGAUUUUUUUGAUUUUUUGGGUUUUAAGUAGGAGGUGUUAGAAAAGUUAUUAUAGGGAUAAUUGGUUUGUGGUGGUUAAGUGUUUAUAG**CG**A**CG**U**CG**UUUUUUGAUUUUU**CG**AUGUUGGUUUUUUUUAUUAUUGUGAAGUAGAAUUUAUUAAGUGUUGGAUUGUUUAUUUAUUAAUAGGGAAUGUGAGUUGGGUUUAGAU **3′**

***Note:*** Do not convert methylated cytosines in the sequence.

55. Determine the sequence of cDNA from bisulfite treated 28S rRNA.
  a. Generate primers for PCR amplification and pyrosequencing using this sequence.

***Note:*** See an example of human 28S rRNA.

**5′**GGGGTTTTATGATTTTTTTGATTTTTTGGGTTTT*AAGTAGGAGGTGTTAGAAAAGTTATTATAGGGATAATTGGTTTGTGGTGGTTAAGTGTTTATAG****CG****A****CG****T****CG****TTTTTTGATTTTT****CG****ATGTTGGTTTTTTTTATTATTGTGAAGTAGAATTTATTAAGTGTTGGATTGT*TTATTTATTAATAGGGAATGTGAGTTGGGTTTAGAT **3′**

***Note:*** Highlighted region as italic is selected for primer design in the next step using PyroMark Assay Design 2.0.

### An example for primer design for 28S rRNA using PyroMark Assay Design 2.0

**Timing: 30 min**

Here, we generate forward and reverse primers, with the latter being biotinylated, to use cDNA generated from bisulfite treated RNA as template. These primers are employed to amplify regions within RNA containing methylated cytosine using classic PCR. Finally, we need a forward or reverse primer, selection is based on contingent upon the biotinylated primers. These are referred to as “sequencing primers” for pyrosequencing, facilitating the sequencing of PCR product amplified from cDNA synthesized from bisulfite converted RNA.

56. In the PyroMark Assay Design 2.0 software with default settings,
  a. Click file,
  b. Select new Methylation Analysis (CpG).
57. Copy cDNA sequence generated from bisulfite treated 28S rRNA into the “Original Sequence Editor” as upper strand field (5′ to 3′).
58. In the “graphic view” section, select the region interested containing CpG sites (here between 80-133).
  a. Right click and select target region.
  b. Select “Set target region”.
  c. Press “Run assay design (F5)”.
59. Generate the primers including forward, reverse and sequencing primers.
60. Select the primers with high score without any warning errors.

***Note:*** The primers generated for human 28S rRNA in this example for the target region are below.

F: 5′ AGTAGGAGGTGTTAGAAAAGT 3′.

R: Biotin-5′ ACAATCCAACRCTTAATAAATTCTACTTC 3′.

Sequencing Primer: 5′ AAGTTATTATAGGGATAATTGG 3′.

**CRITICAL:** Check the original RNA sequencing, if the forward primer contains CpG site, then use “Y” instead of “C” in the primers. In case of reverse primer, use “R” instead of “C” if the primer contains CpG site.

### An example for assay and primer design using PyroMark Assay Design 2.0 for *Homo sapiens* (human) tRNA-Asp (anticodon GTC)

**Timing: 60 min**

Here, we generate primers for Homo sapiens (human) tRNA-Asp (anticodon GTC) 2-1 (TRD-GTC2 1 to 11) | URS00006174C2.

61. Obtain original tRNA-Asp (anticodon GTC) sequence from rnacentral.org.

***Note:*** See the sequence of tRNA-Asp (anticodon GTC) below.

**5′** UCCUCUUAGUAUAGUGGUGAGUAUCCCCGCCUGUCACGCGGGAGACCGGGGUUCGAUUCCCCGACGGGGAG **3′**

62. Determine tRNA-Asp sequence after bisulfite treatment.

***Note:*** See the sequence of tRNA-Asp (anticodon GTC) below after bisulfite treatment.

**5**′UUUUUGUUAGUAUAGUGGUGAGUAUUUU**CG**UUUGUUA**CGCG**GGAGA**CCG**GGGUUUGAUUUUUUGAUGGGGAG**3′**

***Note:*** Do not convert methylated cytosines (target cytosines) in the sequence.

63. Determine cDNA sequence of bisulfite treated tRNA-Asp.
  a. Generate primers for PCR amplification and pyrosequencing using the sequence below

***Note:*** See the sequence of cDNA of tRNA-Asp (anticodon GTC) below.

**5′** *TTTTTGTTAGTATAGTGGTGAGTATTTT****CG****TTTGTTA****CGCG****GGAGA****CCG****GGGTTTGATTTTTTGATGGGG*AG **3′**

***Note:*** “Cs” highlighted in bold are expected to be methylated.

***Note:*** Highlighted region as italic is selected for primer design in the next step using PyroMark Assay Design 2.0.

64. By using PyroMark Assay Design software, determine primers (forward and reverse- biotinylated) for PCR amplification and sequencing primer for pyrosequencing.

***Note:*** The primers below were generated using PyroMark Assay Design software for human tRNA-Asp (GTC) with 94 score and without any warnings.

F: 5′ TTTTTGTTAGTATAGTGGTGAGTATT 3′

R: Biotin-5′ CCCCATCAAAAAATCAAACC 3′

Sequencing Primer: 5′ AGTATAGTGGTGAGTATTT 3′

### PCR amplification

**Timing: 135 min**

Here, we amplify cDNA to obtain enough copy of region interested containing methylated cytosines to measure methylation level by pyrosequencing.

65. Prepare PCR mixture below (Table 5).

**Table 5:** PCR master mix for amplifying target region

Reagent	Amount (μL)
GoTaq Hot Start Green Master Mix, 2X	12.5
F Primer (10 pM)	0.5
R Primer (10 pM)	0.5
Bisulfite Converted cDNA	3
Nuclease-Free Water	8.5
Final Volume	25
66. Pipette 22 μL of PCR mixture to PCR tube, including a no-template control in the layout.
67. Pipette 3 μL of bisulfite converted cDNA to PCR plate.
68. Add 3 μL of water with the same volume of template to the no-template control well.
69. Run PCR with the appropriate conditions.

***Note:*** The volume of bisulfite converted cDNA can be increased for efficient pyrosequencing.

**CRITICAL:** We suggest using gradient PCR approach to determine the exact annealing temperature for each gene set.

***Note:*** We determined 58°C as the most appropriate annealing temperature of primers that we use for PCR amplification for 28S rRNA assay.

***Note:*** We determined 45°C as the most appropriate annealing temperature of primers that we used for PCR amplification for tRNA-Asp (anticodon GTC) assay.

***Note:*** We used 45 cycles for PCR reaction.

### Electrophoresis

**Timing: 45 min**

Here, we check the PCR product, primer dimers, and by products.

70. For 28S rRNA assay, prepare 1.5% agarose gel by mixing 1.5 g agarose with 100 ml 1xTAE buffer on low heat hot plate.
71. After removing from microwave oven add 5 μl of GelGreen.
72. Use 2 μl of 100 bp DNA ladder and 2 μl of samples.
73. Run at 90–100 voltages for 30–40 minutes.

***Note:*** We suggest using higher agarose concentration and low molecular weight DNA ladder for the tRNA assay.

### Processes for pyrosequencing

#### CpG assay design for 28S rRNA using PyroMark Q48 Autoprep Software

**Timing: 30 min**

We perform pyrosequencing to measure m5C level in non-coding RNAs. This example is to import the assay designed by the Assay design software 2.0.

74. Use PyroMark Q48 Autoprep Software to set up a New CpG assay.
75. Save assay design file, which is generated by PyroMark Assay Design 2.0.
76. Select file in the PyroMark Q48 Autoprep software.
77. Select import, then select “Create new CpG assay from assay design file”.
78. Select saved assay by PyroMark Assay Design 2.0.
79. Select orientation as Forward or Reverse.
80. Press the button of “Generate Dispensation Order” to obtain Dispensation Order.
81. Ensure that the software adds a “**C**” to check the efficiency of bisulfite conversion. Save assay file with appropriate name.

***Note:*** In case of DNA, software adds “C” for bisulfite conversion normally. In case of RNA, since we generate cDNA, software does not add “C” for bisulfite conversion.

**CRITICAL:** Control “**C**” for bisulfite treatment can also be added manually.

***Note:*** Assay design can be performed manually by pasting sequence to “Sequence to Analyze” field in the PyroMark Q48 Autoprep Software, and then press generate dispensation order.

#### CpG/CpN assay design for tRNA-Asp using PyroMark Q48 Autoprep Software

**Timing: 30 min**

We present the details about the assay design for human tRNA-Asp. Here, we provide the details by copying and pasting the sequence into the software.

82. Use PyroMark Q48 Autoprep Software to set up a New CpG assay for tRNA-Asp.
83. Click on “New Assay” in the toolbar and select “New CpG Assay”.
84. Copy and enter the sequence to “Sequence to Analysis in pyrosequencing” field.

***Note:*** Alternatively, sequence to analysis can be imported from the Assay design software 2.0.

85. Select CpN mode, since the assay contain one “C” which is not as CpG.
86. Select orientation as Forward or Reverse.
87. Press the button of “Generate Dispensation Order” to obtain Dispensation Order.
88. Saved the file with an appropriate name.

**CRITICAL:** Control “**C**” for bisulfite treatment can be added manually in appropriate position.

***Note:*** In case of non CpG cytosine, CpN mode of the software measures the methylation of cytosine.

***Note:*** Assay design can also be performed as indicated in the previous section “CpG assay design for 28S rRNA using PyroMark Q48 Autoprep software”.

### Prepare for a new run for 28S rRNA-disc setup

**Timing: 30 min**

Here, we set up the sample name and the assay for pyrosequencing in PyroMark Q48 Disc.

89. Use PyroMark Q48 Autoprep Software to set up a New CpG assay run.
90. Click on “New Run” icon in the toolbar.
91. In the disc setup, referring to 48 wells, e.g., select well A1, then select the assay that is set up in previous section (the assay for 28S rRNA).
92. Set up the wells required the sample name that are analyzed.

***Note:*** Add control sample(s) without template in each run.

***Note:*** Add a control containing annealing buffer and the primer

### Perform pyrosequencing

**Timing: 90 min**

93. Turn on the PyroMark Q48 Autoprep and insert absorber strip.
94. Apply cleaning process according to the instruction of machine.
95. Select the disk set up file designed previously.
96. Put all the reagents according to the volume suggested by the machine.

***Note:*** The reagents volume is determined by the machine depending on the number of samples and sequence analyzed in the assay.

***Note:*** Sequencing primer can be added manually, if primer loading is selected as manual in the “Run Set up”

97. Insert a PyroMark Q48 disc into the instrument.

***Note:*** There are two options; the disk can be loaded with template and bead mix. After insertion or outside of the machine.

98. Vortexing the magnetic beads for homogeneity.
99. Add 3 μl of PyroMark Q48 magnetic beads into the required wells of the PyroMark Q48 Disc.

***Note:*** Since the beads precipitate quickly, pipetting of beads should be done immediately after suspension.

100. Adding 10 μL of biotinylated PCR product into the wells of the PyroMark Q48 disc, which has magnetic beads.

**CRITICAL:** Check that the disc is correctly inserted into the machine and locked well.

101. Close injectors cover and press “start” to begin the run.
102. Apply cleaning process after each run.

***Note:*** If manual primer loading is selected, the instrument will estimate the time to add 2 μL of 4 μM sequencing primers to the wells. Therefore, the instrument should be monitored closely to ensure timely manual addition of the primers.

## Expected outcomes

Theoretically, although the RNA-m5C-pyroseq is tested for a single m5C methylation level in 28S rRNA and tRNAs by following this protocol, it can be technically extended to all types of RNAs by developing appropriate assays. In addition, the approach can be used for all types of RNAs isolated for various organisms. At the current stage of epitranscriptomics era, we can generate transcriptome-wide mapping of RNA modifications, providing information about the existence and location of any modification. However, this does not enable us to understand the dynamic changes of modification levels at site-specific individual transcripts or non-coding RNAs. Although the comprehensive sequencing approach has provided extensive data, most importantly, we need to know the biological impact of specific RNA modifications in specific RNA at a specific position. In addition, such approach allows for precise measurement of m5C level, enabling insight into dynamic changes of individual modifications within a particular RNA region in response to cellular, developmental, environmental and diseases.^12^

### Starting amount of RNA

The protocol utilizes a minimal quantity of RNAs, which enables us to employ the protocol for large cohorts in high throughput efforts. Current approaches for comprehensive analysis of sequencing require large amount of RNAs; which are mainly limited to cell lines. This protocol starts with 1000 ng of total RNAs or 1000 ng of small RNAs to perform single nucleotide resolution analysis to quantitatively measure RNA m5C. Such an amount of RNA is manageable from any biological source such as blood or tissues.

### Bisulfite conversion efficiency

The protocol combines bisulfite treatment and pyrosequencing approach, the latest enables us to control bisulfite conversion. Complete bisulfite conversion efficiency is critical since unconverted unmethylated cytosine will be read by pyrosequencing as methylated cytosine, resulting in false positive findings. For example, while the first human cell m5C methylome identified 10,275 sites in mRNAs and other non-coding RNAs,^2^ subsequent investigations in two different mouse cell types reported about 7,500 m5C sites in 1,650 mRNAs in embryonic cells, and 2,075 sites in 486 mRNAs in the brain.^13^ Similarly, around 3,600 m5C sites were detected in 2,000 mRNAs in HeLa cells.^14^ It was suggested that inadequate and unequal bisulfite treatment, the complex structure of RNAs, unpredictable other factors, and limitations related to bioinformatics approaches may all contribute to inconsistent findings, hindering a comprehensive understanding of m5C profiling across RNAs.^15^

**Figure 1:**
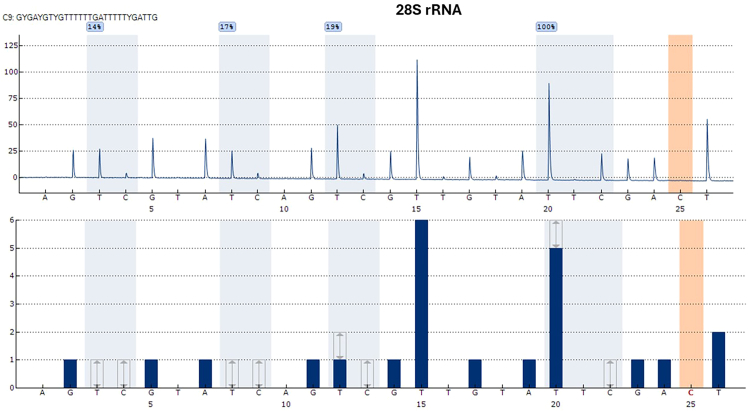
A pyrosequencing result of 28S rRNA We prepared five technical replicates, which were measured three different times. We proved that RNA-m5C-pyroseq potentially measure the methylation in human 28S rRNA at position 4447 as 100%, which is supposed to be 100%. Here, we obtained reproducibility the 100% methylation at last CpG position. Pyrosequencing has also an internal quality assessment indicating three colors; blue indicates that quality of reads reaches high quality, yellow indicates that reads need to be checked, red indicates that reads are failed. As a quality assessment, our reads for 28S rRNA for positive control CpG and three other CpG sites have high quality. As far as we know, there are no reports about the first three CpG sites that we included in 28S rRNA assay. Thus, RNA-m5C-pyroseq has potential to measure low level of methylation, enabling to identify methylated cytosine with quantity with other cytosines. There is a control “C” for bisulfite conversion at the position of 25, in which no peak occurred. It indicates that bisulfite conversion in the assay is 100% efficient.

**Figure 2:**
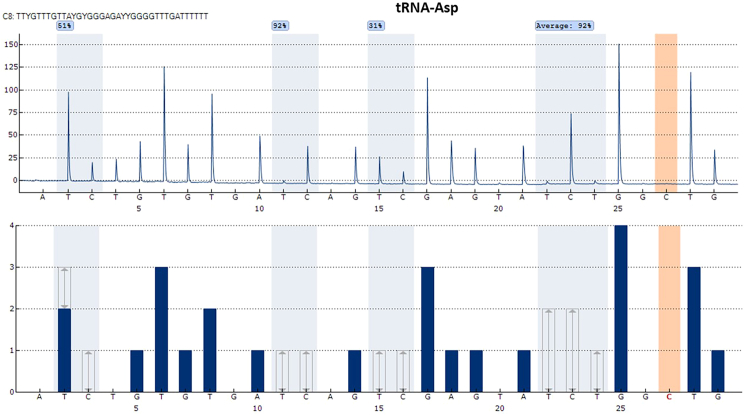
A pyrosequencing result of tRNA-Asp We have prepared two technical replicates, which contains five “C” for methylation analysis. The reads for each time reach high quality, assessed by pyromark software indicating with blue color. In addition, the coefficient of variation (CV) calculating for each reads indicates that CV of each cytosines is lower than 10% indication of high reproducibility. The program generated from tRNA-ASP assay contains bisulfite control “C” at the position of 27. No peak in this position indicates a successful bisulfite conversion for tRNA-ASP assay.

100% efficiency of bisulfite conversion enables us to obtain reproducible results. Although it may depend on the RNA types, we suggest increasing the number of bisulfite conversion cycles for complete efficiency. On the other hand, higher number of bisulfite conversion cycles may result in degradation or reduce the concentration of RNA after bisulfite treatment. One of the advantages of the protocol is to measure the bisulfite efficiency by adding a control “C” to the pyrosequencing. In the current protocol, we measure that bisulfite conversion efficiency is 100%, when we convert total RNA and small RNAs (Figures 1 and 2) as detected by pyrosequencing.

### cDNA synthesis using bisulfite-converted RNA molecules

Following this protocol generates 25 μl of cDNA synthesized from bisulfite converted total RNAs or small RNAs. In case of applying triplicates in pyrosequencing, the protocol use 4.5 μl of 25 μl of cDNA. In total, the total cDNA can be used for approximately 5 different assays designed for different regions of the same RNA molecule or for different RNAs. Using 3 μl of cDNA as template DNA generates 25 μl of PCR product, which is sufficient to run duplicates (10 μl for each run) in pyrosequencing.

### RNA m5C level in four CpG sites in human 28S rRNA

We show the application and efficiency of the protocol by designing two assays including a region containing an approximately 100% methylated CpG site and three CpGs in human 28R rRNA, and an assay of tRNA that contain methylated cytosines in different nucleotides. We obtained a 100% peak for each replicate with high quality pass in the CpG sites as we select as positive control (Figure 1). In addition, we determine the methylation level of three CpG sites, which have not been reported previously. Moreover, we calculate coefficient variation (CV) of repeated measurements, indicating high potential of reproducibility (Table 6).

**Table 6:** Results of technical replicates for 28S rRNA

	CpG1 (%)	CpG2 (%)	CpG3 (%)	CpG4 (expected as 100%)
Mean (n=15)	13.5	12.75	13.69	100
SD	0.75	0.68	1.02	0
CV	0.06	0.05	0.07	0

SD: Standard deviation, CV: Coefficient variation

### RNA m5C level in human tRNA-Asp

Similarly, we perform pyrosequencing for tRNA-Asp assay in triplicates, and we find consistent results between measurements (Table 7). We provide data of two technical replicates, which are measured in different batches. Here, we aim to measure the methylation level of four CpG sites and one C site (Figure 2). We also obtained high reproducibility for the assay of tRNA-Asp. Here, by using the protocol, we can provide robust measurement of methylation level in different methylated cytosine in tRNA-Asp in pre-adipocytes.

**Table 7:** Results of technical replicates for tRNA-Asp

	CpG1 (%)	CpG2 (%)	CpG3 (%)	C+ CpG4 (%)
Mean (n=5)	47.73	91.21	30.17	91.82
SD	3.11	1.48	1.65	0.97
CV	0.07	0.02	0.05	0.01

SD: Standard deviation, CV: Coefficient variation

## Limitations

This protocol is designed with the following objectives: **1)** to achieve efficient bisulfite conversion of RNAs with low starting amount, **2)** to conduct successful cDNA synthesis with bisulfite converted RNAs, **3)** to enable successful generation of a high-quality PCR product for pyrosequencing, and **4)** to produce high-quality pyrosequencing data to measure m5C level at single nucleotide resolution in non-coding RNAs.

One of the limitations is that the RNA is quite sensitive and easily degraded under chemical treatment. It is well-known that treatment with bisulfite also degrades RNA. In our methodology, we measure that the RNA recovery after bisulfite treatment is around >80%. Based on our experiences, the remaining RNA after bisulfite treatment is sufficient to run the experiment successfully. Here, we do not measure the integrity of RNAs after bisulfite treatment, whether degradation of RNAs affect the subsequent experimental process. However, when using >200 ng of bisulfite treated RNA for cDNA synthesis, we do not observe any limitation to obtain cDNA successfully.

In the RNA-m5C-pyroseq, assay design including three types of primer sets is a critical part of the methodology. Due to the nature of the methodology including bisulfite treatment following cDNA generation and pyrosequencing in the final step, each assay requires specific designing of primers for each step. Furthermore, due to the complex secondary structure of RNAs, insufficient denaturation processes may inhibit the amplification of the target region and decrease bisulfite conversion. Thus, for each assay or RNA type, the RNA denaturation stage might need to be tested to establish suitable conditions and ensure complete bisulfite conversion efficiency.

Following bisulfite conversion, the generation of cDNA from bisulfite-treated RNA can lead to homopolymer regions, such as poly-T stretches. This issue has been noted in previous reports, where pyrosequencing can resolve homopolymers of 3-5 nucleotides. In our experience, homopolymers consisting of 5 consecutive thymine (T) bases may result in inaccurate measurement of the expected peak heights. This technical challenge can be addressed by designing a new set of primers that avoids targeting methylation sites in those regions.

Troubleshooting regarding pyrosequencing and reagents are well documented in the PyroMark Q48 Autoprep User Manual.

## Troubleshooting

The primary goal of this protocol is to ensure successful bisulfite conversion of RNA, successful cDNA synthesis from bisulfite converted RNA, successful PCR amplification and the generation of high-quality pyrosequencing data. The protocol is tested by measuring methylation levels in a CpG site, which was previously reported as 100%.^2^^,^^4^ Successful tests are conducted for human 28S rRNA and tRNA-Asp. However, there are several critical points for successful pyrosequencing.

### Problem 1

m5C in small RNAs (Step 17–25).

Due to the low amount of small RNAs in total RNA, measuring m5C level in small RNAs, such as tRNAs may not possibly start with 1000 ng of total RNA.

### Potential solution

We suggest using small RNA-enriched fractions enrichment step or purified from total RNAs, which yield successful PCR product for pyrosequencing.

### Problem 2

Low efficiency of bisulfite conversion (Step 34).

Pyrosequencing checks the bisulfite conversion efficiency. Due to secondary structure of RNAs, bisulfite conversion may not be complete.

### Potential solution

• Apply denaturation cycle with 70°C before bisulfite conversion.
• Increase the number of bisulfite treatment cycle including denaturation step, which enable to increase bisulfite efficiency.

### Problem 3

Low or no yield of cDNA or PCR product (Step 17–25).

Low level of some RNAs, such as tRNAs, in total RNA.

### Potential solution

Isolate small RNAs.

### Problem 4

Low or no yield of cDNA (Step 47).

The concentration of bisulfite treated RNA is not enough (>200 ng) or primer selection.

### Potential solution

• Increase the concentration of RNA.
• Use random hexamer, specific primers, or combination of hexamer and specific primers.

### Problem 5

Low yield or byproducts after PCR (Step 69).

Primer design.

### Potential solution

Run gradient PCR to find exact annealing temperature.

### Problem 6

Byproducts or primer dimers (Step 73).

Proper annealing temperature or condition.

### Potential solution

Check the annealing temperature or generate new primers.

### Problem 7

Small peaks in control dispensations in pyrosequencing (Step 102).

Contamination of pyrophosphate.

### Potential solution

Clean nucleotide injectors with pyrophosphatase.

### Problem 8

Low light signal in pyrosequencing (Step 102).

Low concentration of PCR product.

### Potential solution

Check the primer, annealing temperature, and cycle number.

## Resource availability

### Lead contact

Further information and requests for resources and reagents should be directed to and will be fulfilled by the lead contact, Yvonne Böttcher (yvonne.bottcher@medisin.uio.no).

### Technical contact

Technical questions on executing this protocol can be directed to and will be answered by Akin Cayir (akin.cayir@medisin.uio.no).

### Materials availability

This study did not generate any new materials.

### Data and code availability

This study did not generate new code. Original data are shown in the figures. This work did not generate any additional datasets.

## Acknowledgments

This work was supported by a research grant awarded by UNIFOR-FRIMED-102372157 to Y.B. and A.C.

## Author contributions

A.C. optimized the protocol, wrote the manuscript., and performed the experiments. Y.B. supervised the work and provided resources and funding. All authors reviewed and edited the manuscript.

## Declaration of interests

The authors declare no competing interests.
